# Synthesis, Characterizations, and 9.4 Tesla T_2_ MR Images of Polyacrylic Acid-Coated Terbium(III) and Holmium(III) Oxide Nanoparticles

**DOI:** 10.3390/nano11051355

**Published:** 2021-05-20

**Authors:** Shanti Marasini, Huan Yue, Son Long Ho, Ji Ae Park, Soyeon Kim, Ki-Hye Jung, Hyunsil Cha, Shuwen Liu, Tirusew Tegafaw, Mohammad Yaseen Ahmad, Adibehalsadat Ghazanfari, Kwon-Seok Chae, Yongmin Chang, Gang Ho Lee

**Affiliations:** 1 Department of Chemistry, College of Natural Sciences, Kyungpook National University, Taegu 41566, Korea; shantimarasini.sm@gmail.com (S.M.); yuehuan888@gmail.com (H.Y.); sonlongh@gmail.com (S.L.H.); liushuwen0701@gmail.com (S.L.); tirukorea@gmail.com (T.T.); yaseen.knu@gmail.com (M.Y.A.); adibeh.ghazanfari@gmail.com (A.G.); 2 Division of RI-Convergence Research, Korea Institute of Radiological and Medical Science (KIRAMS), Seoul 01817, Korea; jpark@kirams.re.kr (J.A.P.); ksy0188@kirams.re.kr (S.K.); 3 Medical Device-Bio Research Institute, Korea Testing and Research Institute, Gwacheon 13810, Korea; kihyessi@ktr.or.kr; 4 Department of Molecular Medicine, School of Medicine, Kyungpook National University, Taegu 41944, Korea; hyunsil901002@gmail.com; 5 Department of Biology Education, Teacher’s College, Kyungpook National University, Taegu 41566, Korea; kschae@knu.ac.kr

**Keywords:** Tb_2_O_3_, Ho_2_O_3_, nanoparticles, polyacrylic acid-coating, high MR field, MRI

## Abstract

Polyacrylic acid (PAA)-coated lanthanide oxide (Ln_2_O_3_) nanoparticles (NPs) (Ln = Tb and Ho) with high colloidal stability and good biocompatibility were synthesized, characterized, and investigated as a new class of negative (T_2_) magnetic resonance imaging (MRI) contrast agents at high MR fields. Their r_2_ values were appreciable at a 3.0 T MR field and higher at a 9.4 T MR field, whereas their r_1_ values were negligible at all MR fields, indicating their exclusive induction of T_2_ relaxations with negligible induction of T_1_ relaxations. Their effectiveness as T_2_ MRI contrast agents at high MR fields was confirmed from strong negative contrast enhancements in in vivo T_2_ MR images at a 9.4 T MR field after intravenous administration into mice tails.

## 1. Introduction

Biomedical imaging is a rapidly growing field in nanomedicine [1,2,3]. Among various imaging techniques, magnetic resonance imaging (MRI), which detects proton spin relaxation signals is highly sensitive and its image spatial resolution is very high because of ample protons in living objects [1,2]. T_2_ MRI detects transverse proton spin relaxation signals and the image contrast becomes darker in the presence of contrast agents because transverse proton spin relaxations are accelerated by the contrast agents. The excellent physicochemical properties of nanoparticles (NPs) make them suitable for use as advanced imaging agents in diagnosing diseases [4,5,6,7,8,9,10]. Among the NPs, lanthanide oxide (Ln_2_O_3_) NPs can play an important role in biomedical imaging because of their exceptionally large paramagnetic moments at room temperature, making them suitable for use as MRI contrast agents even at ultrasmall particle sizes [8,9,10]. Such Ln_2_O_3_ NPs include those with Ln as gadolinium (Gd) (^8^S_7/2_), terbium (Tb) (^7^F_6_), dysprosium (Dy) (^6^H_15/2_), holmium (Ho) (^5^I_8_), and erbium (Er) (^4^I_15/2_), which all possess high atomic magnetic moments [11].

Paramagnetic Ln_2_O_3_ NPs have unsaturated magnetization (M) at clinical MR fields (H) at room temperature [12,13]. Therefore, their M values will increase with increasing H until their M values become saturated. Notably, their saturated M values are even larger than those of iron oxide NPs [12,13], implying that they can be extremely powerful MRI contrast agents at high H [12,13,14,15,16]. Ultrasmall NPs with a particle diameter (d) <3 nm are known to be eligible for renal excretion [17,18,19]. Therefore, the ultrasmall Ln_2_O_3_ NPs will be extremely valuable as MRI contrast agents.

According to the outer sphere model, a high M value is critical for negative (T_2_) MRI contrast agents because the transverse water proton relaxation is accelerated by the fluctuation of local magnetic fields generated by contrast agents [20,21,22]. Therefore, NPs are the ideal choice for T_2_ MRI contrast agents because small molecules do not have such high M values. The iron oxide NPs with ultrasmall particle diameters become less powerful as T_2_ MRI contrast agents because of their reduced M values at ultrasmall particle diameters [23] because their M values rapidly drop with decreasing particle diameter [24], contrary to the virtually particle size-independent M values of Ln_2_O_3_ NPs. This is likely because electron spins in diffuse 3d-orbitals are largely affected by surface-coating ligands whereas those in compact 4f-orbitals are not: this can be noticed from the small ligand field splitting of ~100 cm^−1^ of Ln(III) ions, whereas that of 3d-transition metal ions is ~20000 cm^−1^ [25]. In addition, bulk materials of Ln_2_O_3_ NPs are also paramagnetic [26,27,28], and thus their M values are nearly size-independent up to bulk materials.

It is critical that Ln_2_O_3_ NPs are tightly grafted with hydrophilic and biocompatible ligands to ensure their good colloidal stability and biocompatibility for in vivo applications [29,30]. Polymers seem to be better surface-coating ligands than small molecules because polymers have abundant hydrophilic groups for binding to NPs whereas small molecules only have a few such groups. For example, polyacrylic acid (PAA) is biocompatible [31] and has abundant COOH groups as hydrophilic and binding groups (i.e., one COOH group per monomer unit). Therefore, PAA has been used as a surface-coating ligand for biomedical applications [32,33,34,35]. Here, the multiple COOH groups on each PAA polymer can make strong electrostatic bonds with a NP and thus each PAA can be tightly grafted on a NP surface. In addition, polymethyl vinyl ether-alt-maleic acid (PMVEMA) [36] and polyacrylic acid-co-maleic acid (PAAMA) [37] can also provide excellent colloidal stability, but PAA was used here because its size and structure are smaller and simpler than those of PAAMA and PMVEMA and thus seems to be more suitable as a coating ligand of ultrasmall NPs.

Herein, we report the one-pot polyol synthesis of PAA-coated Ln_2_O_3_ NPs (Ln = Tb and Ho) with high colloidal stability and good biocompatibility. The synthesized PAA-coated NPs were subject to various analyses and their effectiveness as T_2_ MRI contrast agents at high MR fields was demonstrated by taking in vivo T_2_ MR images in mice at a 9.4 T MR field.

## 2. Materials and Methods

### 2.1. Chemicals

Tb(NO_3_)_3_ × 5H_2_O (99.9%), Ho(NO_3_)_3_ × 5H_2_O (99.9%), NaOH (>98%), triethylene glycol (TEG) (99%), PAA (M_w_ = ~1800 Da), sodium acetate buffer solution (3.0 M, pH = 7.0), fetal bovine serum (FBS), and Roswell Park Memorial Institute (RPMI) 1640 medium were purchased from Sigma Aldrich, St. Louis, MO, USA, and used as-received. C_2_H_5_OH (99%) was purchased from Duksan Chemical Co., Ansan, South Korea, and used as-received for the initial washing of the PAA-coated Ln_2_O_3_ NPs (Ln = Tb and Ho). Triple-distilled water (Pure Power I+, Human Co., Seoul, Korea) was used for the final washing of the PAA-coated Ln_2_O_3_ NPs and in preparing their suspension samples.

### 2.2. One-Pot Polyol Synthesis of PAA-Coated Ln_2_O_3_ NPs (Ln = Tb and Ho)

**Figure 1:**
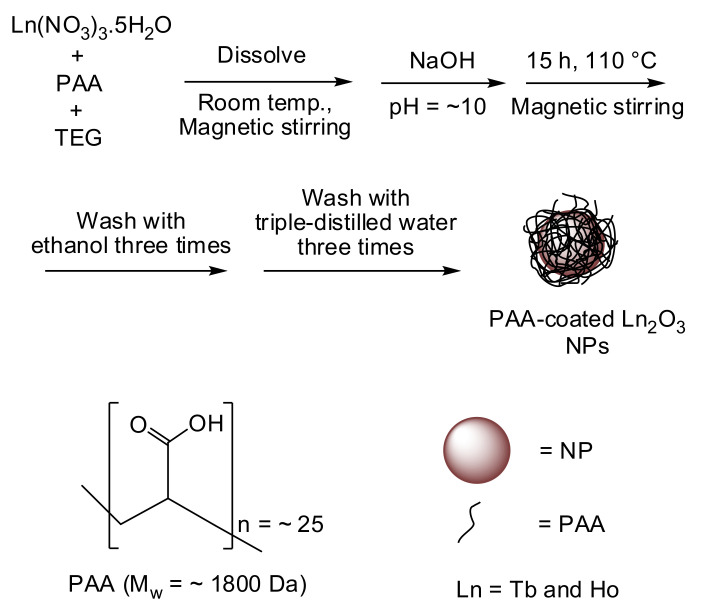
One-pot polyol synthesis of the PAA-coated Ln_2_O_3_ NPs (Ln = Tb and Ho).

To synthesize the PAA-coated Ln_2_O_3_ NPs (Figure 1), 2 mmol of Ln^3+^-precursor salt and 2.0–2.5 mmol of PAA were added to 20 mL of TEG in a three-necked round-bottom flask. The mixture solution was magnetically stirred at room temperature under atmospheric conditions until the precursor salt dissolved in the TEG. In a separate beaker, a NaOH solution consisting of 10 mmol of NaOH in 15 mL of TEG was prepared. The NaOH solution was slowly added to the precursor solution until the pH of the mixture solution reached 10. At high pH values (pH > 8.0), more stable Ln(OH)_3_ is mainly formed rather than the Ln^3+^–PAA complex [38]. The Ln(OH)_3_ is then further converted into Ln_2_O_3_ NPs at elevated temperatures [39]. After the pH became nearly constant, the reaction temperature was slowly increased to 110 °C using a heating oil bath and maintained at that temperature for 15 h with constant magnetic stirring. The reaction mixture was then cooled to room temperature and transferred to a 500-mL beaker. The obtained PAA-coated NPs were washed with 400 mL of ethanol three times to remove Na^+^, OH^−^, TEG, PAA, and the unreacted precursors. The product solution was diluted with 400 mL of ethanol and then stored in a refrigerator (~4 °C) for several days until the product NPs precipitated. The upper transparent portion of the solution was decanted and the remaining product solution was washed with ethanol again. To remove ethanol from the product NPs, 400 mL of triple-distilled water was added to the product solution, which was then concentrated to a volume of 20–30 mL using a rotary evaporator that removed the ethanol. This washing process was repeated three times. The production yield was 60–70%. This good yield and low production cost suggest that the present method is economically effective. The product solution was split into two parts: one half volume was subject to a powder sample by freeze-drying it under vacuum, and the other half volume was diluted with triple-distilled water to prepare a suspension sample.

### 2.3. General Characterizations

The particle diameter of the PAA-coated Ln_2_O_3_ NPs (Ln = Tb and Ho) was measured using a high-resolution transmission electron microscope (HRTEM) (Titan G2 ChemiSTEM CS Probe, FEI, Hillsboro, OR, USA) operated at 200 kV acceleration voltage. Each sample dispersed in ethanol was dropped onto a carbon film supported by a 200-mesh copper grid (Pelco No. 160, Ted Pella Inc., Redding, CA, USA) using a micropipette (Eppendorf, 2–20 μL). The copper grid with the sample was allowed to dry in air for an hour at room temperature before mounting inside the HRTEM for measurement. The Ln-concentration in each suspension sample was measured using an inductively coupled plasma atomic emission spectrometer (ICPAES) (IRIS/AP, Thermo Jarrell Ash Co., Waltham, MA, USA). Each sample was pretreated with HCl/HNO_3_ (1:3 mole ratio) acids to completely dissolve NPs in solution before measurement. The hydrodynamic diameter was measured using a dynamic light scattering (DLS) particle size analyzer (Zetasizer Nano ZS, Malvern, Malvern, UK). For measurement, the NP suspension samples with 0.01 mM Ln were used. The zeta potentials (Zetasizer Nano ZS, Malvern, Malvern, UK) were measured using the NP suspension samples with 0.01 mM Ln. The colloidal stability was also investigated by measuring the transmission (T) and backscattering (BST) of thee near infrared (NIR) beam (880 nm) as a function of h (h = 5 to 10 mm from the vial bottom containing the sample solution) and t for t = 0 to three days using a Turbiscan (Turbiscan AGS, Formulaction, Paule Raymondis, France). The crystal structure of the powder samples before and after thermogravimetric analysis (TGA) was measured using a multi-purpose x-ray diffraction (XRD) spectrometer (X’PERT PRO MRD, Philips, The Netherlands) with unfiltered CuKα radiation (λ = 1.54184 Å) and 2θ = 15–100°. A Fourier transform-infrared (FT-IR) absorption spectrometer (Galaxy 7020A, Mattson Instrument Inc., Madison, WI, USA) was used to investigate the surface coating of the NPs. For measurement, pellets of powder samples in KBr were prepared. The amount of PAA-coating on the NP surfaces was estimated by recording TGA curves (SDT-Q600, TA Instrument, New Castle, DE, USA) using powder samples between room temperature and 900 °C while air flowed over the powder sample. The amount of PAA-coating was quantified in wt.% from the mass drop in the TGA curve after taking into account the initial mass drop between room temperature and ~105 °C due to water desorption. A vibrating sample magnetometer (VSM) (7407-S, Lake Shore Cryotronics Inc., Westerville, OH, USA) was used to characterize the magnetic properties of the powder samples by recording the magnetization (M) versus applied field (H) (or M−H) curves (−1.8 T ≤ H ≤ 1.8 T) at 300 K. The measurements were carried out using powder samples of 20–30 mg. The net M value of each sample (i.e., only the Ln_2_O_3_ NPs without the PAA coating) was estimated using the net mass of the Ln_2_O_3_ NPs extracted from the TGA curve.

### 2.4. In Vitro Cellular Cytotoxicity Measurements

The in vitro cellular toxicity of the PAA-coated Ln_2_O_3_ NPs (Ln = Tb and Ho) was assessed using a CellTiter-Glo Luminescent Cell Viability Assay (Promega, Madison, WI, USA). In this assay, a luminometer (Synergy HT, BioTek, Winooski, VT, USA) was used to quantify intracellular adenosine triphosphate. Human prostate cancer (DU145) and normal mouse hepatocyte (NCTC1469) cell lines were used as test cells. These two cell lines were used because it would be better to use one cancer and one normal cell line rather than to use two cancer or two normal cell lines. In addition, they are common cell lines. Both cells were seeded on a 24-well cell culture plate and incubated for 24 h (5 × 10^4^ cell density, 500 μL of cells per well, 5% CO_2_, and 37 °C). Five test NP suspension samples were prepared by diluting each concentrated NP suspension sample with a sterile phosphate-buffered saline solution. Then, 2 μL of each test suspension sample was added to the cells to have 10, 50, 100, 200, and 500 μM Ln in the treated cells, and the treated cells were incubated for 48 h. The viabilities of the incubated cells were measured and normalized with respect to the control cells with 0.0 M Ln. The measurements were repeated in triplicate to obtain the average cell viabilities.

### 2.5. Relaxometric Property Measurements

To obtain longitudinal (r_1_) and transverse (r_2_) water proton spin relaxivities, longitudinal (T_1_) and transverse (T_2_) water proton spin relaxation times were measured using a 3.0 T MRI scanner (MAGNETOM Trio Tim, Siemens, Munchen, Germany) and a 9.4 T MRI scanner (9.4 T MRI System, Varian, Palo Alto, CA, USA). A series of NP suspension samples (1, 0.5, 0.25, 0.125, 0.0625, and 0.0 mM Ln) (Ln = Tb and Ho) were prepared by diluting the original concentrated NP suspension samples with triple-distilled water. T_1_ relaxation times were measured using an inversion recovery method. T_2_ relaxation times were obtained using a multiple spin-echo method with a Carr–Purcell–Meiboom–Gill pulse sequence. Then, the r_1_ and r_2_ values were estimated from the slopes in the plots of 1/T_1_ and 1/T_2_ versus Ln-concentration, respectively.

### 2.6. In Vivo T_2_ MR Image Measurements

In vivo animal imaging studies were performed in accordance with the rules and regulation and permission of the animal research committee of the Korea Institute of Radiological and Medical Science. A 3.0 T MRI scanner (MAGNETOM Trio Tim, Siemens, Munchen, Germany) and a 9.4 T MRI scanner (9.4 T MRI System, Varian, Palo Alto, CA, USA) were used to obtain the in vivo T_2_ MR images. Two or three Institute of Cancer Research (ICR) mice weighing ~30 g were used for each sample. The mice were anesthetized using 1.5% isoflurane in oxygen. Measurements were made before and after the injection of a NP suspension sample prepared in triple-distilled water into mice tail veins. The injection dose was approximately 0.1 mmol Ln/kg (Ln = Tb and Ho) (injection volume = 70 μL). During measurements, body temperature of the mice was maintained at 37 °C using a warm water blanket. After measurements, the mice were revived from anesthesia and placed in a cage with free access to food and water. The spin echo sequence was used to obtain T_2_ MR images. The typical measurement parameters were as follows: H = 3.0 (or 9.4) T, echo time (TE) = 30 ms, repetition time (TR) = 2000 ms, frequency encoding step = 320 Hz, phase = 320, number of acquisitions (NEX) = 4, field of view (FOV) = 70 mm, slice thickness = 1.0 mm, and slice gap = 1.1 mm.

## 3. Results

### 3.1. Particle Diameter (d), Hydrodynamic Diameter (a), Zeta Potential (ξ), and Crystal Structure

**Figure 2:**
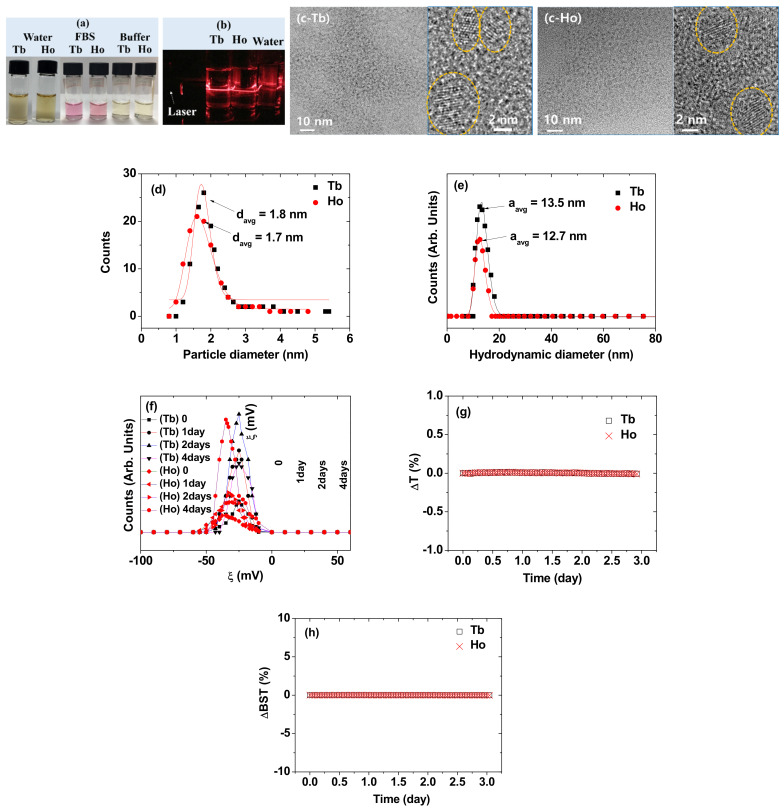
(**a**) Photographs of nanoparticle suspension samples in aqueous media, 10% FBS in RPMI1640 medium, and sodium acetate buffer solution (pH = 7.0), (**b**) laser light scattering (i.e., Tyndall effect) proving NP dispersions: laser light scattering was observed only for both solution samples whereas no light scattering was observed for the reference triple-distilled water, (**c**) HRTEM images at low (10 nm scale) and high (2 nm scale) magnifications: dotted circles indicate the PAA-coated Ln_2_O_3_ NPs (Ln = Tb and Ho), (**d**) particle diameter distributions and log-normal function fits (the total number of NPs, N_total_ = 133 for PAA-coated Tb_2_O_3_ NPs and 111 for PAA-coated Ho_2_O_3_ NPs), (**e**) hydrodynamic diameter distributions and log-normal function fits, (**f**) plots of zeta potentials (ξ) overtime (0, 1, 2, and 4 days) [insets are plot of ξ versus time (t)], (**g**) plots of ΔT [= T(t) − T(0); T = height-averaged transmission of NIR beam] as a function of time, and (**h**) plots of ΔBST [= BST(t) − BST(0); BST = height-averaged backscattering of NIR beam] as a function of time. Labels “Tb” and “Ho” indicate the PAA-coated Tb_2_O_3_ and Ho_2_O_3_ NPs, respectively.

The estimated Ln-concentrations of the solution samples using an ICPAES were 28.42 mM Tb for the PAA-coated Tb_2_O_3_ NPs and 30.44 mM Ho for the PAA-coated Ho_2_O_3_ NPs. PAA has abundant COOH groups (Figure 1) and thus can be tightly grafted on a Ln_2_O_3_ NP surface through many electrostatic bonds between its COOH groups and the many Ln^3+^ on the NP surface. Consequently, the NP suspension samples showed good colloidal stability, as shown in the NP suspension samples in Figure 2a (i.e., the NPs had not precipitated since they were prepared (>one year)). The colloidal stability was additionally checked in a 10% FBS in RPMI 1640 medium and a sodium acetate buffer solution (pH = 7.0). The NPs were stable in these media (i.e., no precipitation for 20 days) (Figure 2a). The observed good colloidal stability in 10% FBS in RPMI1640 medium was consistent with the fact that PAA negligibly interacts with proteins such as albumin at physiological pH [40]. Each vial containing the NP suspension sample exhibited laser light scattering (i.e., Tyndall effect) due to the collision of the NP suspension with laser light whereas no light scattering was observed for the vial containing the triple-distilled water (Figure 2b), proving the good colloidal dispersion of the NPs in aqueous solutions.

HRTEM imaging was used to estimate the particle diameters of the PAA-coated Ln_2_O_3_ NPs (Ln = Tb and Ho) (Figure 2c). The core Ln_2_O_3_ NPs could be clearly seen through lattice fringes in HRTEM images (see images at 2 nm scale), but the shell PAA coating around the NP core could not be seen because it is almost impossible to see small polymers like PAA through HRTEM imaging. The remaining is the background amorphous carbon covering the copper grid onto which the NPs were dispersed. As can be seen in the HRTEM images, the NPs were ultrasmall and nearly spherical. Their average particle diameters (d_avg_) were estimated to be 1.8 ± 0.1 nm for the PAA-coated Tb_2_O_3_ NPs and 1.7 ± 0.1 nm for the PAA-coated Ho_2_O_3_ NPs from log-normal function fits to the observed particle diameter distributions (Figure 2d and Table 1). As can be seen in Figure 2d, most of the NPs were less than 3 nm in diameter, making them amenable to renal excretion [17,18,19], while some of them were larger than 3 nm, but less than 5.5 nm. In addition, the PAA-coated Tb_2_O_3_ NPs were slightly bigger than the PAA-coated Ho_2_O_3_ NPs. DLS was used to estimate the hydrodynamic diameters of the PAA-coated Ln_2_O_3_ NPs (Figure 2e). Applying log-normal function fits to the observed DLS patterns, the average hydrodynamic diameters (a_avg_) were estimated to be 13.5 nm for the PAA-coated Tb_2_O_3_ NPs and 12.7 nm for the PAA-coated Ho_2_O_3_ NPs (Figure 2e and Table 1). For both samples, the difference between a_avg_ and d_avg_ was approximately 11 nm, indicating large hydration spheres for both samples and thus explaining the observed good colloidal stability for both samples. The zeta potentials (ξ) were measured overtime (Figure 2f). The observed highly negative and constant ξ values over time (inset in Figure 2f) supported the excellent colloidal stability of the NP solution samples. The average ξ values are provided in Table 1. Here, the negative surface charges of NPs can be balanced by Na^+^ in solution because as described in the experimental section, the PAA-coated Ln_2_O_3_ NPs were produced in basic solutions (pH = ~10) using NaOH. The colloidal stability of the PAA-coated Ln_2_O_3_ NPs was also confirmed through the transmission (T) and backscattering (BST) measurements of NIR beam as a function of time (t) for three days. The ΔT (t) and ΔBST (t), corresponding to average T (t) and average BST (t) minus average T (t = 0) and average BST(t = 0), respectively, in which the average T (t) and average BST (t) are the averages of transmitted and backscattered NIR beam intensities, respectively, for all NIR beam heights (h) (h was changed between 5 and 10 mm from the vial bottom containing the sample solution) at a scan time t, were plotted as a function of t (Figure 2g,h, respectively). Note that both the ΔT (t) and ΔBST (t) are zero for stable colloids. Therefore, the negligible deviations of ΔT (t) and ΔBST (t) from zero for both solution samples confirmed the stable colloidal suspensions for both solution samples. Therefore, all results confirmed the excellent colloidal stability for both solution samples. The leaching experiments of Tb^3+^ and Ho^3+^ ions from the samples were carried out by dispersing 4.5 mg of powder samples in 4.0 mL of triple-distilled water and dialyzing them against 1.5 L of triple-distilled water (MWCO = 1000 amu) for two days. After dialysis, the solutions outside the dialysis bag were concentrated to ~1.8 mL using a rotary evaporator. The measured metal ion concentration was below the detection limit of the ICPAES for both samples, indicating negligible leaching of metal ions from the samples.

**Figure 3:**
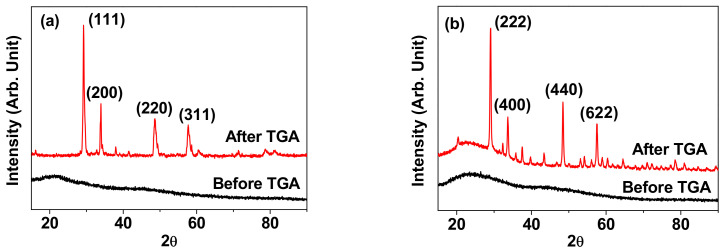
XRD patterns before (bottom spectra) and after TGA (top spectra) of the PAA-coated Ln_2_O_3_ NPs [Ln = (**a**) Tb and (**b**) Ho]. Only the intense peaks were representatively assigned with (hkl) Miller indices for the XRD patterns after TGA.

The crystal structures of both powder samples before and after TGA were determined by XRD analysis. Both samples showed very broad and amorphous XRD patterns before TGA (bottom XRD patterns in Figure 3a,b), indicating that most of the NPs were not fully crystallized due to their ultrasmall size [39]. After TGA, however, sharp peaks corresponding to cubic structure of bulk materials were observed for both samples due to particle size growth and crystallization (top XRD patterns in Figure 3a,b). All peaks after TGA could be assigned with (hkl) Miller indices but only intense peaks were representatively assigned in the XRD patterns. The estimated cell constants of 5.28 Å for the Tb_2_O_3_ NPs and 10.61 Å for the Ho_2_O_3_ NPs after TGA were consistent with previously reported values [41].

### 3.2. Surface-Coating Results

**Figure 4:**
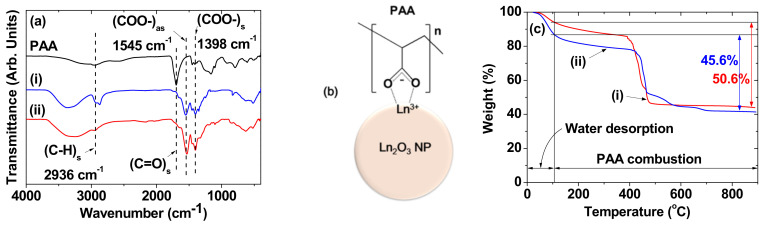
(**a**) FTIR absorption spectra of the powder samples and free PAA, (**b**) bridge-bonding structure of COO^−^ with Ln^3+^ on a NP surface (many such bridge-bonding structures exist per NP because of many COO^−^ groups per PAA and many Ln^3+^ on a NP surface), and (**c**) TGA curves of the powder samples: the numbers are the amounts of PAA-coating in wt.%. Labels (i) and (ii) indicate the PAA-coated Ln_2_O_3_ NPs [Ln = (i) Tb and (ii) Ho].

The surface coating was examined by FTIR absorption spectroscopy using powder samples. Characteristic absorption peaks of PAA [42] such as the C–H stretch at 2936 cm^−1^ and the C=O stretch at 1697 cm^−1^ were observed in the FTIR absorption spectra of the samples (Figure 4a). However, the C=O stretches for PAA in the samples were red-shifted by ~154 cm^−1^ from that of free PAA. This is due to electrostatic bonding of the COO^−^ groups in PAA to Ln^3+^ (Ln = Tb and Ho) on the NP surfaces, which has been observed previously in various molecules with carboxylic groups bonded to metal oxides [43,44]. The two peaks in the sample spectra at 1555–1535 cm^−1^ and 1395–1400 cm^−1^ with a frequency difference of ~147 cm^−1^ were the asymmetric and symmetric stretching frequencies of COO^−^, respectively, due to the bridge-bonding of the COO^−^ of PAA with Ln^3+^ on a NP surface [45], as schematically drawn in Figure 4b. Because each PAA (M_w_ = ∼1800 Da) has ∼25 carboxylic groups, it is expected that many of them per PAA were conjugated to each NP. Because NaOH was used in the synthesis, free (or unconjugated) carboxylic groups of PAA in the PAA-coated NPs were in the form of COO^−^Na^+^ (not COOH), which may appear at 1555 cm^−^^1^ [46], slightly higher than that (= ∼1545 cm^−^^1^) of conjugated COO^−^ to Ln^3+^ of the NP. Therefore, it was difficult to see the peaks of free carboxylic groups (i.e., COO^−^Na^+^) of PAA in the PAA-coated NPs due to their overlap with the peaks of COO^−^ conjugated to Ln^3+^ of the NP.

The average amount (P) of PAA-coating on the NP surface was estimated in wt.% from the mass drop in the TGA curves (Figure 4c). The initial mass drop between room temperature and ~105 °C was attributed to water desorption. The following mass drop (i.e., P) was due to the combustion of PAA due to its oxidation reaction with flowing hot air. The remaining mass was due to Ln_2_O_3_ NPs (Ln = Tb and Ho). The average number of PAA polymers coating a unit NP surface area (i.e., grafting density (σ) [47]) was calculated using the molecular mass of PAA (= ~1800 Da), the d_avg_ estimated from HRTEM imaging, and the bulk density (7.91 g/cm^3^ for Tb_2_O_3_ and 8.41 g/cm^3^ for Ho_2_O_3_ [48]). The average number (N_PAA_) of PAA polymers coating the NP surface was estimated by multiplying σ by the NP surface area (πd_avg_^2^). The surface-coating results are summarized in Table 1.

### 3.3. In Vitro Cellular Cytotoxicity Results

**Figure 5:**
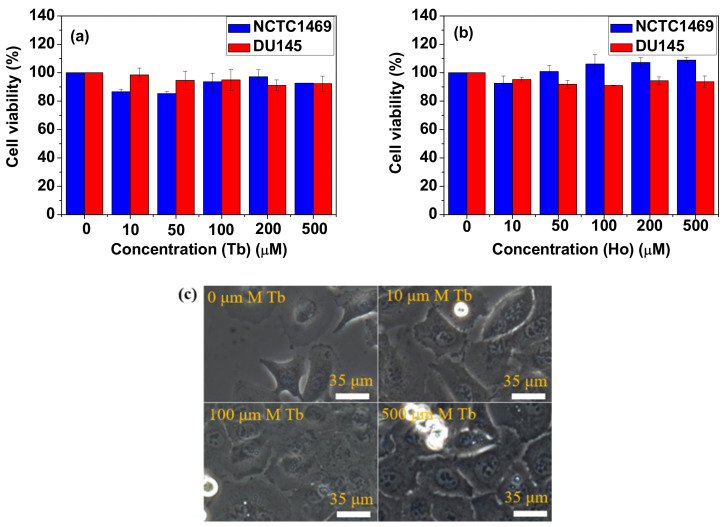
In vitro cell viabilities of NCTC1469 and DU145 cells incubated with the PAA-coated Ln_2_O_3_ NPs [Ln = (**a**) Tb and (**b**) Ho]. (**c**) Optical images of DU145 cancer cells incubated with PAA-coated Tb_2_O_3_ NPs at various Tb-concentrations.

The in vitro cell viabilities of both DU145 and NCTC1469 cells treated with NP suspension samples were greater than 90% up to a 500 μM Ln-concentration for both samples (PAA-coated Tb_2_O_3_ NPs in Figure 5a and PAA-coated Ho_2_O_3_ NPs in Figure 5b), showing very low cellular toxicities for both samples. The mice also survived after in vivo MRI experiments (>1 year), proving good biocompatibility of both samples. The optical images of DU145 cancer cells incubated with PAA-coated Tb_2_O_3_ NPs at various Tb-concentrations (Figure 5c) showed that the NPs seemed to be more concentrated at the cells and cell nuclei with increasing Tb-concentration.

### 3.4. Magnetic Properties

**Figure 6:**
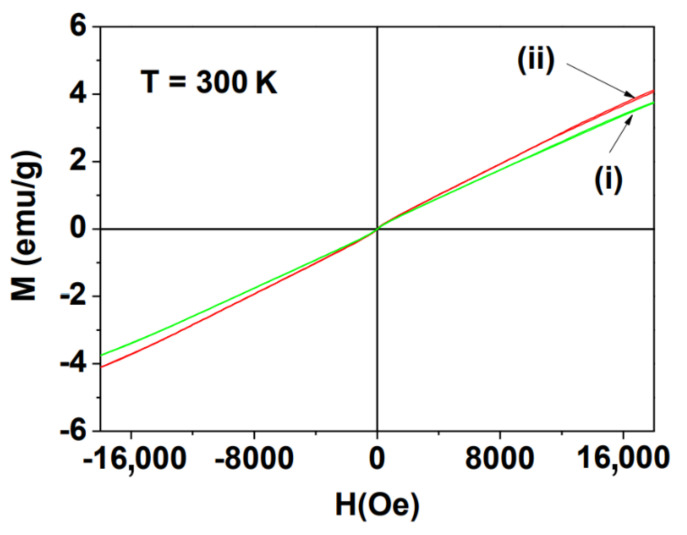
M−H curves of the PAA-coated Ln_2_O_3_ NPs at 300 K [Ln = (i) Tb and (ii) Ho]. Net M values of the Ln_2_O_3_ NPs (i.e., without PAA) estimated from TGA curves were used in the plots.

The magnetic properties of the powder samples were characterized by recording the M versus H (or M−H) curves (−1.8 T ≤ H ≤ 1.8 T) at 300 K (Figure 6). Both samples were paramagnetic (i.e., no hysteresis, zero coercivity, and zero remanence in the M-H curves), similar to their corresponding bulk materials [26,27,28]. The measured M value of each sample was mass-corrected using the net mass of the NPs [i.e., the mass of the Ln_2_O_3_ (Ln = Tb and Ho) only, without PAA] estimated from the TGA curves. From the mass-corrected M−H curves, the net M values at 1.8 T were estimated to be 3.8 emu/g for the Tb_2_O_3_ NPs and 4.1 emu/g for the Ho_2_O_3_ NPs (Table 1). The slightly higher M value (in emu/g) of the Ho_2_O_3_ NPs than that of the Tb_2_O_3_ NPs is due to the higher atomic magnetic moment (μ = 10.60 μ_B_) of Ho than that (= 9.72 μ_B_) of Tb [11], in which μ_B_ is the Bohr magneton.

### 3.5. r_1_ and r_2_ Values

**Figure 7:**
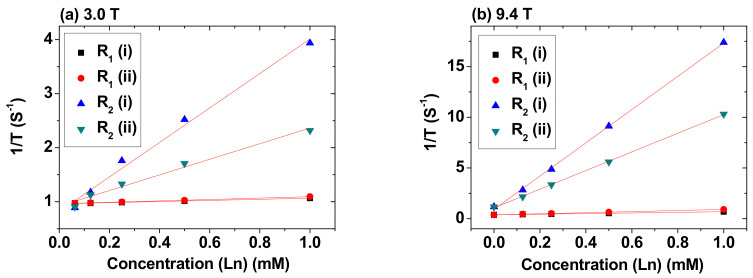
Plots of 1/T_1_ and 1/T_2_ versus Ln-concentration at (**a**) 3.0 T and (**b**) 9.4 T. Labels (i) and (ii) indicate the PAA-coated Ln_2_O_3_ NPs [Ln = (i) Tb and (ii) Ho].

The r_1_ and r_2_ water proton spin relaxivities were estimated from the slopes in the plots of inverse T_1_ and T_2_ water proton spin relaxation times (i.e., 1/T_1_ and 1/T_2_) versus Ln-concentration (Ln = Tb and Ho), respectively, at H = 3.0 T (Figure 7a) and 9.4 T (Figure 7b). Both samples showed negligible r_1_ values at all H, but their r_2_ values were appreciable at H = 3.0 T and higher at H = 9.4 T (Table 1).

### 3.6. In Vivo T_2_ MR Images at 9.4 T MR Field

**Figure 8:**
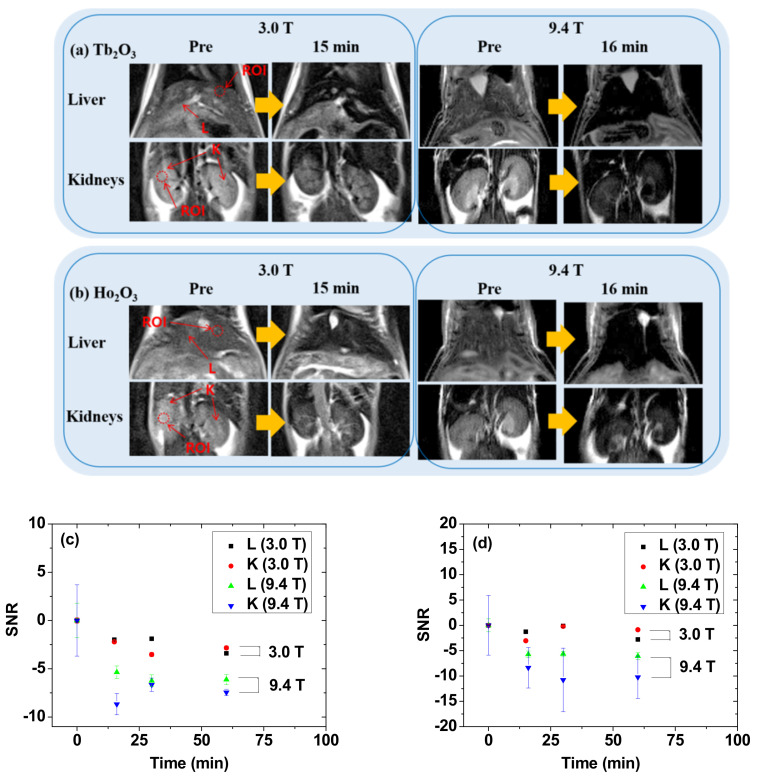
In vivo T_2_ MR images of the liver and kidneys at 3.0 and 9.4 T MR fields before (=Pre) and 15 (or 16) min after intravenous administration of aqueous suspension samples of the PAA-coated Ln_2_O_3_ NPs into mice tails [Ln = (**a**) Tb and (**b**) Ho]. SNR plots of ROI as a function of time (0 = Pre) at 3.0 and 9.4 T MR fields for the PAA-coated Ln_2_O_3_ NPs [Ln = (**c**) Tb and (**d**) Ho]. Labels at each first MR image on the left: ROI = small dotted circles; L = liver; K = kidney.

The effectiveness of both samples as T_2_ MRI contrast agents at high MR fields was examined by taking in vivo T_2_ MR images at a 9.4 T MR field (right images in Figure 8a,b). In addition, in vivo T_2_ MR images at 3.0 T MR field were also taken for comparison (left images in Figure 8a,b). Each NP suspension sample prepared in triple-distilled water was intravenously administered into mice tails and T_2_ MR images were acquired before and after administration. The T_2_ MR images acquired 15 (or 16) min after administration showed negative contrast enhancements (i.e., darker images) in the liver and kidneys in both samples with respect to those before administration. The negative contrast enhancements were appreciable at a 3.0 T MR field and stronger at a 9.4 T MR field for both samples. The stronger negative contrast enhancements at a 9.4 T MR field than those at a 3.0 T MR field for both samples were attributable to their higher r_2_ values at a 9.4 T MR field than those at a 3.0 T MR field (Figure 7 and Table 1). These stronger negative contrast enhancements at a 9.4 T MR field can be quantitatively seen in the plots of signal-to-noise ratio (SNR) of a region-of-interest (ROI) of the liver and kidneys with respect to those before administration (Figure 8c,d). These strong negative contrast enhancements at a 9.4 T MR field confirm that both samples are potential T_2_ MRI contrast agents at high MR fields. Considering that ultrasmall NPs (d < 3.0 nm) are amenable to renal excretion [17,18,19], these results imply that the PAA-coated Ln_2_O_3_ NPs (Ln = Tb and Ho) might be applied to the detection of diseases such as cancer at an early stage, which could be explored in the future. 

## 4. Discussion

PAA-coated Ln_2_O_3_ (Ln = Tb and Ho) NPs were synthesized through the one-pot polyol method (Figure 1). Compared to D-glucuronic acid as a surface-coating ligand [49], PAA enhanced the colloidal stability as well as biocompatibility of the NPs.

The observed negligible r_1_ and appreciable r_2_ values can be explained as follows. The magnitudes of the r_1_ and r_2_ values depend on the degree of T_1_ and T_2_ water proton spin relaxations induced by the NPs, respectively. According to the inner sphere model, T_1_ water proton spin relaxation is mainly accelerated by the magnetic dipole–dipole interaction between the 4f-electron spins of Ln^3+^ (Ln = Tb and Ho) on the NP surfaces and the water proton spins in contact with or close to the NPs [22,50]. This interaction is negligible because the fast 4f-electron motions of Tb^3+^ and Ho^3+^ do not match with the slow water proton spin motions [50], thus providing negligible r_1_ values. On the other hand, according to the outer sphere model, T_2_ water proton spin relaxation is induced by the fluctuation of local magnetic fields generated by the NPs [20,21]. The model suggests that r_2_ ∝ M_NP_^2^, in which M_NP_ (in emu/NP) is a magnetic moment per NP [20,21,22]. The M_NP_ values of the Ln_2_O_3_ NPs are appreciable at room temperature, as can be noticed from their M values in Figure 6, explaining the observed appreciable r_2_ values at 3.0 T. In addition, the enhanced r_2_ values of the NPs at 9.4 T were due to their higher M_NP_ values at 9.4 T because the paramagnetic NP samples were far from saturation (Figure 6) and consequently, M_NP_ value increase with increasing H until M_NP_ becomes saturated.

As shown in Table 1, bigger r_2_ values were observed for the PAA-coated Tb_2_O_3_ NPs than the PAA-coated Ho_2_O_3_ NPs. This is attributable to slightly larger particle diameters of the PAA-coated Tb_2_O_3_ NPs (d_avg_ = 1.8 nm) than those of the PAA-coated Ho_2_O_3_ NPs (d_avg_ = 1.7 nm). The r_2_ value of lanthanide oxide NPs is very sensitive to the particle diameter because bigger NPs can have larger M_NP_ values. The M_NP_ can be approximately estimated assuming a spherical shape for NPs and using d_avg_ as follows: M_NP_ ≈ μ × [number (n) of Ln^3+^ per NP] and n ≈ (2/5) × (d_avg_/w)^3^, in which w is the average ionic diameter of all the atoms in chemical formula [w (Tb_2_O_3_) = 0.236 nm and w (Ho_2_O_3_) = 0.234 nm using d (O^2−^) = 0.252 nm, d (Tb^3+^) = 0.2126 nm, and d (Ho^3+^) = 0.2082 nm] [51,52]. Therefore, M_NP_ (Tb_2_O_3_ NP)/M_NP_ (Ho_2_O_3_ NP) ≈ [μ (Tb) × (d_avg_/w)^3^ (Tb_2_O_3_ NP)]/[μ (Ho) × (d_avg_/w)^3^ (Ho_2_O_3_ NP)] = [9.72 × (1.8/0.236)^3^]/[10.60 × (1.7/0.234)^3^] = 1.1. Therefore, M_NP_ (Tb_2_O_3_ NP) > M_NP_ (Ho_2_O_3_ NP), which explains the observed bigger r_2_ values of the PAA-coated Tb_2_O_3_ NPs than those of the PAA-coated Ho_2_O_3_ NPs.

**Figure 9:**
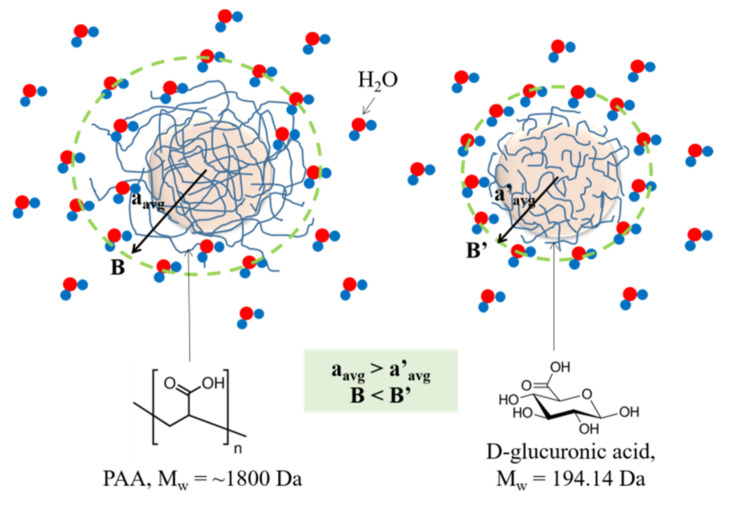
Scheme explaining the ligand-size effects on hydrodynamic diameter and r_2_ value. a_avg_ and a_avg_’ = average hydrodynamic diameter; B and B’ = magnetic field generated by the NP.

The observed r_2_ values were compared with other values (Table 2) [49,53,54]. Compared to r_2_ values of D-glucuronic acid-coated Ln_2_O_3_ NPs (Ln = Tb and Ho) [49] with similar particle diameters as those of PAA-coated Ln_2_O_3_ NPs, the PAA-coated NPs exhibited lower r_2_ values due to their larger coating ligands, explained as follows. The hydrodynamic diameters (a) of the PAA-coated Ln_2_O_3_ NPs (Ln = Tb and Ho) were approximately twice those [49] of the D-glucuronic acid-coated Ln_2_O_3_ NPs at the similar core particle diameters and the r_2_ values of the PAA-coated Ln_2_O_3_ NPs were smaller than those [49] of the D-glucuronic acid-coated Ln_2_O_3_ NPs. These surface-coating ligand effects are schematically explained in Figure 9. PAA can accommodate more water molecules through its long chain length and many carboxylic groups (∼25 groups per PAA) than small D-glucuronic acid: therefore, each PAA-coated NP can be swollen into a bigger size than each D-glucuronic acid-coated NP, thus allowing a larger hydrodynamic diameter. However, D-glucuronic acid with a smaller size than PAA can allow more water molecules to closely approach the NP, making more water molecules feel stronger magnetic fields (B) generated by the NP, consequently making the D-glucuronic acid-coated NPs have a higher r_2_ value. On the other hand, large NPs and nanorods [53,54] exhibited high r_2_ values even with a large ligand coating because of their enhanced paramagnetic magnetic moment per NP or per nanorod, which increases with increasing particle size and because the r_2_ value is proportional to the square of the magnetic moment [14].

## 5. Conclusions

PAA-coated Ln_2_O_3_ NPs (Ln = Tb and Ho) (d_avg_ = 1.8 and 1.7 nm, respectively) were prepared via one-pot polyol synthesis, and investigated as a new class of T_2_ MRI contrast agents at high MR fields.

(1) Both samples exhibited excellent colloidal stability and good biocompatibility resulting from PAA-coating on the NP surfaces.
(2) The appreciable r_2_ values at a 3.0 T MR field (3.19 s^−1^∙mM^−1^ for the PAA-coated Tb_2_O_3_ NPs and 1.44 s^−1^∙mM^−1^ for the PAA-coated Ho_2_O_3_ NPs), enhanced r_2_ values at a 9.4 T MR field (16.40 s^−1^∙mM^−1^ for the PAA-coated Tb_2_O_3_ NPs and 9.20 s^−^1∙mM^−1^ for the PAA-coated Ho_2_O_3_ NPs), and negligible r_1_ values at all MR fields for both samples, indicated their exclusive induction of T_2_ relaxations with negligible induction of T_1_ relaxations at all MR fields, and a stronger induction of T_2_ relaxations at a higher MR field.
(3) The strong negative contrast enhancements in the in vivo T_2_ MR images of mice at a 9.4 T MR field confirmed the effectiveness of the NPs as T_2_ MRI contrast agents at high MR fields.

## Figures and Tables

**Table 1:** Particle diameter (d), hydrodynamic diameter (a), zeta potential (ξ), surface-coating amount (P, σ, N_PAA_), magnetic properties, and water proton spin relaxivities (r_1_, r_2_) of the PAA-coated Ln_2_O_3_ NPs (Ln = Tb and Ho).

NP	Particle Dimension		ξ_avg_ (mV)	Surface-Coating Amount			Net M ^4^ (emu/g)	Water Proton Spin Relaxivity (s^−1^∙mM^−1^)			
	d_avg_ (nm)	a_avg_ (nm)		P ^1^ (wt.%)	σ ^2^ (1/nm^2^)	N_PAA_^3^		3.0 T		9.4 T	
								r_1_	r_2_	r_1_	r_2_
Tb_2_O_3_	1.8 ± 0.1	13.5 ± 0.2	−25.0 ± 0.1	50.6 ± 0.2	0.80 ± 0.05	6.4 ± 0.1	3.8 ± 0.1	0.10 ± 0.01	3.19 ± 0.01	0.30 ± 0.01	16.40 ± 0.01
Ho_2_O_3_	1.7 ± 0.1	12.7 ± 0.2	−32.9 ± 0.1	45.5 ± 0.2	0.85 ± 0.05	7.4 ± 0.1	4.1 ± 0.1	0.13 ± 0.01	1.44 ± 0.01	0.53 ± 0.01	9.20 ± 0.01

^1^ Average surface-coating amount of PAA polymers in wt.% estimated from TGA. ^2^ Grafting density (i.e., average number of PAA polymers coating a unit surface area of a NP). ^3^ Average number of PAA polymers coating a NP. ^4^ M at 1.8 T and 300 K (emu/g).

**Table 2:** Comparison of r_2_ values.

Sample	Size (nm)	Coating Ligand	Applied Field (T)	r_2_ (s^−1^∙mM^−1^)	References
Tb_2_O_3_	2.0	D-glucuronic acid	1.5	7.68	[49]
Tb_2_O_3_	2.0	D-glucuronic acid	3.0	33.97	[49]
Tb_2_O_3_	2.0	D-glucuronic acid	9.4	53.67	[49]
Ho_2_O_3_	1.9	D-glucuronic acid	1.5	7.76	[49]
Ho_2_O_3_	1.9	D-glucuronic acid	3.0	35.21	[49]
Ho_2_O_3_	1.9	D-glucuronic acid	9.4	56.33	[49]
Tb_2_O_3_	1.8	PAA, 1800 amu	3.0	3.19	This study
Tb_2_O_3_	1.8	PAA, 1800 amu	9.4	16.40	This study
Ho_2_O_3_	1.7	PAA, 1800 amu	3.0	1.44	This study
Ho_2_O_3_	1.7	PAA, 1800 amu	9.4	9.20	This study
Ho_2_O_3_	67–81	PEG^1^, 4000 amu	1.5	23.47	[53]
Tb-nanorod	9.0 × 2.1	GA-PEG, 3170 amu	1.44	10.5	[54]
Tb-nanorod	9.0 × 2.1	GA-PEG, 3170 amu	9.4	48.5	[54]

PEG = polyethylene glycol. GA = gallic acid.

## Data Availability

The data presented in this study are available on request from the corresponding authors.
